# 2665. Health Care Provider Characteristics Associated with Anal Cancer Screening in an Academic Medical System

**DOI:** 10.1093/ofid/ofad500.2276

**Published:** 2023-11-27

**Authors:** Daniel Gore, Ashley O’Donoghue, Tenzin Dechen, Jessica A Zerillo, Ami Multani, Douglas Krakower

**Affiliations:** Beth Israel Deaconess Medical Center, Boston, Massachusetts; Beth Israel Deaconess Medical Center, Boston, Massachusetts; Beth Israel Deaconess Medical Center, Boston, Massachusetts; Beth Israel Deaconess Medical Center, Boston, Massachusetts; Fenway Health, Boston, Massachusetts; Harvard Medical School, Boston, MA

## Abstract

**Background:**

Anal cancer disproportionally affects persons living with HIV (PWH), sexual and gender minorities, and those with gynecologic cancers, condyloma acuminata, and solid organ transplantation. While expert guidance on anal cancer screening exists for PWH, there are no national guidelines for screening any of these populations. We analyzed clinician characteristics associated with anal cancer screening among providers who care for high-risk populations at an academic medical center including infectious disease (ID) specialists, primary care providers (PCPs), and obstetrician/gynecologists (OBGYNs).

**Methods:**

We retrospectively reviewed medical charts for patients with HIV, gynecologic cancers, condyloma acuminata, solid organ transplantation, and sexual or gender minority status at Beth Israel Deaconess Medical Center and two affiliated clinics from 01/01/2015 - 08/01/2022. We extracted provider specialties, clinic locations, patient panel demographics, and dates of anal cytology and human papillomavirus (HPV) screening tests. We used chi-squared tests to identify provider factors associated with screening.

**Results:**

Of 1,093 providers, at least one anal cytology test was performed by 93.8% (75/80) of ID providers, 55.2% (376/681) of PCPs, 28.3% (41/145) of OBGYNs, and 24.1% (45/187) of other providers (p < 0.001); among screeners, anal HPV co-testing was performed equally across specialties (54.2%, p = 0.23). Providers were more likely to screen if they cared for PWH (91.8% with 10-49 PWH vs 3.7% for no PWH, p < 0.001), majority cis-men (55.2%, p=0.003), or majority publicly insured patients (51.8%, p < 0.001). Providers were less likely to screen if they cared for majority cis-women (40.9%, p< 0.001) or majority privately insured patients (24.0%, p< 0.001). Screening practices did not differ by the racial composition of providers’ patient panels (p=.21).

**Conclusion:**

While many ID specialists performed anal cancer screenings, other providers were less likely to screen. Providers caring for many PWH, cis-men, or publicly insured patients were more likely to screen. Additional research is needed to clarify screening guidance and optimize screening practices for all higher-risk populations, especially non-PWH groups.

**Disclosures:**

**Douglas Krakower, MD**, Gilead: Grant/Research Support|Merck: Grant/Research Support|U. North Texas Health Sciences Center: Funding for mentoring|UAB: Advisor/Consultant|UpToDate, Inc.: Royalties|Virology Education: Honoraria

